# Relationship between biomechanics and energy cost in graded treadmill running

**DOI:** 10.1038/s41598-023-38328-x

**Published:** 2023-07-28

**Authors:** Marcel Lemire, Robin Faricier, Alain Dieterlen, Frédéric Meyer, Grégoire P. Millet

**Affiliations:** 1 Faculty of Sport Sciences, University of Strasbourg, Strasbourg, France; 2 School of Kinesiology, The University of Western Ontario, London, ON Canada; 3 Institut de Recherche en Informatique, Mathématiques, Automatique Et Signal, Université de Haute-Alsace, 68070 Mulhouse, France; 4 Institute of Sport Sciences, University of Lausanne, Lausanne, Switzerland; 5 Digital Signal Processing Group, Department of Informatics, University of Oslo, Oslo, Norway

**Keywords:** Applied physics, Physiology

## Abstract

The objective of this study was to determine whether the relationships between energy cost of running (Cr) and running mechanics during downhill (DR), level (LR) and uphill (UR) running could be related to fitness level. Nineteen athletes performed four experimental tests on an instrumented treadmill: one maximal incremental test in LR, and three randomized running bouts at constant speed (10 km h^−1^) in LR, UR and DR (± 10% slope). Gas exchange, heart rate and ground reaction forces were collected during steady-state. Subjects were split into two groups using the median Cr for all participants. Contact time, duty factor, and positive external work correlated with Cr during UR (all, *p* < 0.05), while none of the mechanical variables correlated with Cr during LR and DR. Mechanical differences between the two groups were observed in UR only: contact time and step length were higher in the economical than in the non-economical group (both *p* < 0.031). This study shows that longer stance duration during UR contributes to lower energy expenditure and Cr (i.e., running economy improvement), which opens the way to optimize specific running training programs.

## Introduction

Although complex physiological and biomechanical factors play important roles in level running (LR), the energy cost of running (Cr) appears as one of the three main predictive factors determining performance^1^. The Cr represents the amount of energy required per unit of kilometer at a given submaximal running velocity allowing to maintain a physiological steady state^2^. Though, the influence of LR Cr on graded running performance remains unclear^3–6^. It has been reported that LR cost of locomotion is a poor indictor of performance in short distance trail races^3,4^ but the importance of energy cost on ultramarathon remains debated^5,6^. A relationship has been observed between the oxygen cost (amount of oxygen consumed per distance unit) in uphill running (UR) and LR in elite ultra-trail runners^7^. However, additional specific parameters such as knee extensor muscle endurance and UR Cr may play a role on inclined running performance^4^.

It has been suggested that changes in the running pattern from negative to positive slopes explain the positive linear increase in Cr with positive slope^8–11^, but not in downhill running (DR), where the relationship between the slopes and Cr has an U-shape with the lowest Cr value at approximately − 10 to − 20% slope^9,12^. While each individual naturally develops their optimal running pattern (i.e., spatiotemporal parameters of stride, running gait) according to their personal characteristics in order to lower their Cr^13,14^, it is well known that changing this self-selected running pattern may alter the Cr^15–18^. Therefore, one may suggest that the most economical runners efficiently adapt their running mechanics to the slope condition. Though, which biomechanical adaptations are associated with a lower Cr remains an open question. According to experimental data, DR involves braking muscle actions of the lower limbs and is considered a predominantly eccentric exercise modality^19^. In contrast, UR predominantly involves concentric propulsive muscle contractions^19^. While the physiological adaptations to hilly terrain are currently being widely investigated, the main performance determinants for LR, UR and DR may differ, with a greater contribution of biomechanical parameters in DR performance^20^.

Compared to LR, UR induces a decrease of aerial time and step length, whereas DR increases the aerial time and the step length. However, the contact phase is less affected by slope, leading to an increase of step frequency during UR^9,10,21,22^. Furthermore, the ratio of positive to negative work is another important biomechanical factor that may explain the slope-dependent variations in Cr^9,23^. The mechanical positive and negative external works (W_ext_^+^ and W_ext_^−^, respectively) represent the work performed at each step to support the upward and downward movements of the center of mass of the body (CoM), respectively^24^. As the downward movement of the CoM decreases with positive slopes, W_ext_^−^ decreases and the W_ext_^+^ increases, and conversely with negative slopes the downward movement increases whereas the upward decreases^24^. Since the energy required to perform W_ext_^−^ is less than that of W_ext_^+^^25^, the more positive the slope, the higher the concentric muscle actions for the elevation of the CoM, leading to an increase in energy expenditure^8^. Conversely, the energy demand in DR is lowered due to the increased part of the eccentric muscle activation and the gravity effect, saving energy^8^. Nevertheless, the direct relationship between running mechanics and Cr in UR or DR is under investigated.

The stride kinematic adaptations in graded running may also influence Cr, as debated in LR^13^. One study has investigated the relationship between running economy and spatiotemporal running parameters within specific slope conditions in a homogeneous group of well-trained runners and reported correlations between spatiotemporal parameters only in DR^12^. The Cr in DR was negatively correlated with both step frequency and step length while positively correlated with contact time^12^. The step length and frequency, and the vertical stiffness were negatively correlated whereas ground contact time was positively correlated with Cr in DR^12^. Conversely, Lussiana et al.^14^ showed that minimal shoes reduced contact time and increased aerial phase whatever the slope condition (± 8% slope), while Cr was not affected. Taken together these results tend to show that biomechanical responses may affect Cr in graded running. However, to the best of our knowledge, no study examined these relationships, including ground reaction forces, in a large heterogeneous group.

Moreover, the running pattern appears to be dependent on the fitness level in LR. Lower vertical forces were observed during the stance phase in a group of runners with the lowest oxygen consumption for a given speed (~ 13 km h^−1^)^17^. The magnitude of peak vertical force determines the work performed by the leg muscles to support the running motion. During incline running, these peak vertical forces have been shown to increase and decrease during DR and UR, respectively^21^. Nevertheless, to our knowledge, no previous study has attempted to determine whether peak vertical forces are also a factor of Cr in incline running (DR and/or UR).

Identification of key running pattern parameters associated with low Cr may have direct practical applications such as developing grade-specific training methods to improve running technique and potentially performance. Thus, it appears interesting to investigate whether specific biomechanical responses can distinguish economical and less economical runners.

Therefore, the objectives of this study were, first, to determine if there was a relationship between Cr and mechanical responses associated with the running pattern; and second, to determine if these biomechanical responses were different between two groups of different Cr levels (economical vs. non-economical). Our hypotheses were first that Cr values would correlate with their biomechanical responses; and second that biomechanical responses would be different between two groups of different economy levels in each slope condition.

## Methods

### Participants

Nineteen volunteer athletes took part in this study (Table 1) and were informed of the benefits and risks of this investigation before giving their written informed consent. They performed between one and five session per week of running training but were not trail specialists. The experiment was previously approved by our Institutional Review Board (CCER-VD 2015-00006) and complied with the Declaration of Helsinki.

**Table 1:** Participant characteristics (*n* = 19).

Age (years)	34	±	10
Height (cm)	175	±	10
Body mass (kg)	68.5	±	12.2
BMI (kg m^−2^)	22.2	±	2.3
vV̇O_2max_ (km h^−1^)	17.3	±	2.3
V̇O_2max_ (mlO_2_ kg^−1^ min^−1^)	58.3	±	7.7
VT1 (mlO_2_ kg^−1^ min^−1^)	40.8	±	4.9
VT2 (mlO_2_ kg^−1^ min^−1^)	54.0	±	7.3
HR_max_ (bpm)	179	±	12

*vV̇O*_*2max*_ velocity associated to V̇O_2max_, *VT1 and VT2* V̇O_2_ at the first and the second ventilatory thresholds, respectively, *HR*_*max*_ maximal heart rate.

### Experimental setup

All participants completed (1) a level running (0% slope) incremental test to exhaustion; and (2) three randomized running bouts at constant velocity (10 km h^−1^) with different slope conditions, LR, UR (+ 10%) and DR (− 10%). The running speed of 10 km h^−1^ was selected to ensure that subjects were below the second ventilatory threshold in each slope condition. Participants performed all the sessions on a treadmill (T-170-FMT, Arsalis, Belgium) at the same time of the day with 1 week of recovery allocated. The subjects were instructed to not perform any eccentric and/or strenuous exercises in this time interval.

### Maximal incremental level running test

The first session was an incremental running test until exhaustion. The test began at 8 km h^−1^ for 4 min and then the speed increased by 1 km h^−1^ every min. During each session, V̇O_2_, carbon dioxide output (V̇CO_2_), and respiratory exchange ratio (RER) were collected breath-by-breath through a facemask with an open-circuit metabolic cart with rapid O_2_ and CO_2_ analyzers (Quark CPET, Cosmed, Rome, Italy) in order to calculate the Cr. Heart rate (HR) was continuously measured (Polar Electro, Kempele, Finland). The highest V̇O_2_ value over 30 s during the maximal incremental test represented the V̇O_2max_. The speed associated with V̇O_2max_ (vV̇O_2max_) was determined as the speed of the step that elicited V̇O_2max_^26^. The first ventilatory threshold was determined as a breakpoint in the plot of V̇CO_2_ as a function of V̇O_2_. At that point, the ventilatory equivalent for O_2_ (V̇_E_/V̇O_2_) increases without an increase in ventilatory equivalent for CO_2_ (V̇_E_/V̇CO_2_)^27^. The second ventilatory threshold was located between the first ventilatory threshold and V̇O_2_max, when V̇_E_/V̇CO_2_ starts to increase while V̇_E_/V̇O_2_ continues to rise^28^. These thresholds were blind assessed by two accustomed experimenters. The average value was kept, and in case of a difference above 30 s, a third experimenter was involved, and the average of the two closest values was used. The rate of perceived exertion was obtained by using a designed scale^29^ to assess the exercise intensity about 30 s after the end of the test. During the second session, after a short warm-up participant performed three randomized constant velocity running bout of 4 min. As for the maximal incremental test, V̇O_2,_ V̇CO_2_ and RER continuously recorded. Before each session, the O_2_ and CO_2_ analyzers were calibrated according to the manufacturer's instructions.

### Metabolic power during constant velocity bouts in level, uphill, and downhill running

Mean Cr values were recorded between 3:15 and 3:45 (min:s) of each running bout. The Cr was computed as following^12^:

$${\text{Cr}} = \Delta {\text{V}}^{ \cdot } {\text{O}}_{2} /(v \times 1000) \times 60 \times E\left( {O_{2} } \right)$$

where Cr is expressed in J kg^−1^ m^−1^, ΔV̇O_2_ for the difference between oxygen consumption at steady-state and oxygen consumption at baseline in mlO_2_ kg^−1^ min^−1^ ^30^, *v* corresponded to the velocity of the trial (10 km h^−1^), and E(O_2_) for O_2_ energy equivalent determined with RER. As the V̇O_2_ response is slope-dependent in running^31^, for each slope condition (i.e., LR, UR and DR), the subjects were arbitrarily divided into two groups (i.e., economical vs. non-economical) based on the absolute Cr median value (2.42, 3.83, and 6.09 J kg^−1^ m^−1^ for DR, LR, and UR, respectively), to obtain equal proportion of runners within each group^17^.

### Biomechanical data collection and processing

An instrumented treadmill equipped with a three-dimensional force platform sampling 1000 Hz was used in this study. To reduce the noise inherent to the treadmill’s vibrations, we first applied, a second order stop-band Butterworth filter with edge frequencies set at 25 and 65 Hz, on the vertical ground reaction force signal. The filter configuration was chosen empirically to obtain a satisfactory reduction of the oscillations observed during flight phases while minimizing its widening effect during ground contact time. Further data analysis was conducted using MATLAB software version R2021a (MathWorks Inc., Natick, MA, USA). The instants of initial contact and terminal contact were identified using a threshold of 7% of bodyweight on the filtered vertical ground reaction force signal^32^, and ~ 80 steps were analyzed for each condition. The contact time (in ms) is the time between initial and terminal contacts of the same leg, the aerial time (in ms) is the time between the terminal contact of one leg and the initial contact of the opposite leg. Duty factor (expressed in %) was computed as the ratio between the contact time and the stride time (i.e., contact time + aerial time). The step frequency (in Hz) is the reciprocal of the time required for one step (time between two consecutive initial contacts). The step length (m) is the quotient of the treadmill belt speed divided by step frequency. Peak vertical ground reaction force (GRF) was computed over the entire stance phase. The W_ext_ was determined using the method proposed by Saibene and Minetti^33^ and is defined as the sum of potential, and horizontal and vertical kinetic works associated with the displacement of the CoM. The W_ext_^−^ and W_ext_^+^ represent the work done due to decelerate and accelerate, respectively, the body’s CoM with respect to the environment. The percentage of negative work is the ratio between the W_ext_^−^ and the total external work. These data were continuously recorded during 30 s between 3:15 and 3:45 (min:s) of each constant velocity running bouts.

### Statistical analysis

Jamovi statistical software (Jamovi 1.6.23, Sydney; Australia) was used for all statistical analyses. All variables were examined for normality using a Shapiro–Wilk. A repeated measures ANOVA was performed to compare the effect of the slope’s condition on the Cr and the biomechanical data, after using Mauchly’s test to assess sphericity. Bonferroni’s correction was applied on the alpha level to account for repeated univariate testing. When significant effects were observed, Bonferroni’s post-hoc tests were used to localize the significant differences. For each condition of slope, scale intercept and Pearson’s product–moment correlation coefficients (*r*) were used to assess the intensity of the relations between Cr and the selected biomechanical variables, with Bonferroni’s multiplicity correction^33^. A one-way ANOVA was used to compare the biomechanical responses on the treadmill between efficiency groups. For all these analyses, data are expressed as mean ± SD and a *p* value inferior to 0.05 was considered statistically significant.

### Ethics approval

This study was performed in line with the principles of the Declaration of Helsinki. Approval was granted by the Ethics Committee by our Institutional Review Board (CCER-VD 2015-00006).

### Consent to participate

Informed consent was obtained from all individual participants included in the study.

## Results

### Cost of locomotion and biomechanics

**Figure 1:**
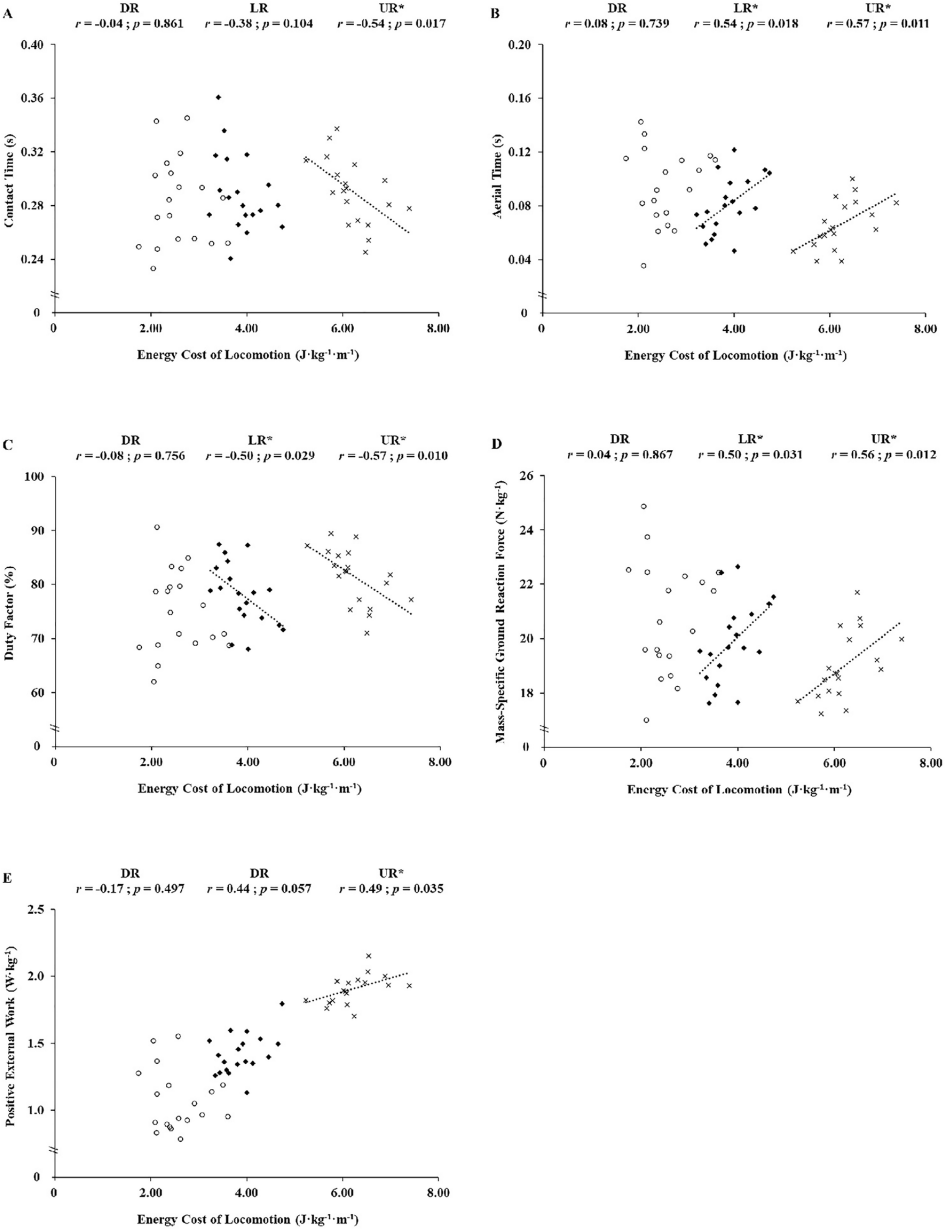
Relationships between the cost of locomotion and contact time (**A**), aerial time (**B**), duty factor (**C**), mass-specific peak vertical ground reaction force (**D**), and positive external mechanical work (**E**) in different slope conditions (*DR* downhill running—unfiled circles, *LR* level running—filled diamonds, *UR* uphill running—*p < 0.05).

Values of Cr and biomechanical parameters in the different slope conditions are presented in Table 2. The contact time was negatively correlated with the Cr in UR only (*r* = − 0.54; *p* = 0.017; Fig. 1). For both UR and LR only, the aerial time was positively correlated (*r* = 0.54 and *r* = 0.57, respectively; both *p* ≤ 0.018; Fig. 1), while the duty factor was negatively correlated with the Cr (*r* = − 0.50 and *r* = − 0.57, respectively; both *p* ≤ 0.029; Fig. 1). The relative peak force was correlated with the Cr in LR and UR only (*r* = 0.50 and *r* = 0.56, respectively; both *p* ≤ 0.031) but not in DR. Regarding mechanical work parameters, the Cr correlated to W_ext_^+^ in UR (*r* = 0.49; *p* = 0.035), but none of the mechanical work parameters correlates with Cr in DR and UR. In addition, Cr was significantly positively correlated between each slope condition: DR-LR (*r* = − 0.57; *p* = 0.011), DR-UR (*r* = − 0.53; *p* = 0.020), and LR-UR (*r* = − 0.72; *p* < 0.001).

**Table 2:** Cost of locomotion and biomechanical parameters in downhill, level and uphill running (*n* = 19).

	Downhill running	Level running	Uphill running	F-STATISTICS
Energy cost of running				
Energy cost (J kg^−1^ m^−1^)	2.56 ± 0.51	3.87 ± 0.43^a^	6.21 ± 0.51^ab^	674
Running kinematics and GRF				
Contact time (s)	0.28 ± 0.03	0.29 ± 0.03	0.29 ± 0.03	3.73
Aerial time (s)	0.09 ± 0.03	0.08 ± 0.02^a^	0.07 ± 0.02^ab^	25.9
Duty factor (%)	74.9 ± 7.6	78.1 ± 5.8^a^	81.5 ± 5.2^ab^	22.6
Step frequency (Hz)	2.66 ± 0.10	2.71 ± 0.12	2.81 ± 0.11^ab^	26.6
Step length (m)	1.05 ± 0.04	1.03 ± 0.05	0.99 ± 0.04^ab^	26.2
Relative step length (% of height)	60.0 ± 3.8	58.8 ± 3.8	56.7 ± 3.3^ab^	25.1
Peak vertical GRF (kN)	1.41 ± 0.20	1.35 ± 0.19^a^	1.29 ± 0.18^ab^	21.8
Mass-specific vertical GRF (N kg^−1^)	20.8 ± 2.1	19.8 ± 1.5^a^	19.0 ± 1.2^ab^	22.1
Mechanical work				
External work (J kg^1^)	3.06 ± 0.44	2.75 ± 0.29^a^	2.76 ± 0.19^a^	17.5
Positive external work (J kg^−1^)	1.07 ± 0.23	1.42 ± 0.15^a^	1.91 ± 0.11^ab^	367
Negative external work (J kg^−1^)	− 1.99 ± 0.22	− 1.34 ± 0.14^a^	− 0.85 ± 0.09^ab^	737
Percentage negative external work (%)	65.3 ± 2.3	48.5 ± 0.2^a^	30.8 ± 1.1^ab^	2270

*GRF* peak ground reaction force.

^a^*p* < 0.05 versus downhill running.

^b^*p* < 0.05 versus level running.

### Economical and non-economical runners

**Figure 2:**
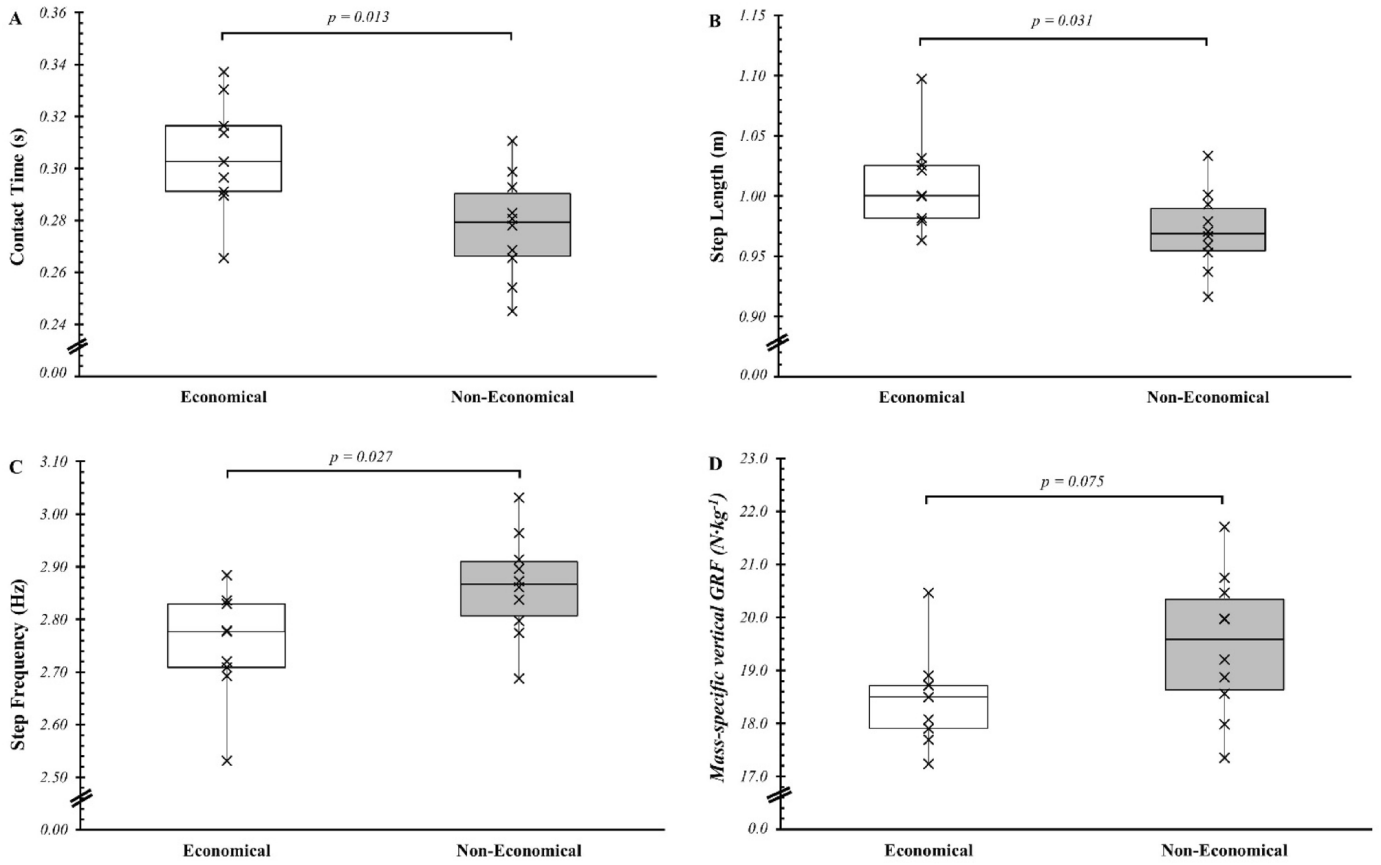
Contact time (**A**), step length (**B**), step frequency (**C**) and mass-specific ground reaction force (**D**) in economical (white box-plots) and non-economical runners (gray box-plots) during uphill running.

All running pattern parameters were similar between economical and non-economical groups in both DR and LR (Table 3). The contact time, step length, and mass-specific peak vertical GRF (Fig. 2A, B, D) were higher while the step frequency (Fig. 2C) was lower in the economical than in the non-economical group in UR (all *p* < 0.031). However, aerial time, GRF, or mechanical work values were not different between the two groups in UR (all *p* > 0.05).

**Table 3:** Comparison of the cost of locomotion and biomechanical responses for economical versus non-economical runners in the three slope conditions (*n* = 19).

	Downhill running			Level running			Uphill running		
	Economical N = 9	Non-economical N = 10	*p*	Economical N = 9	Non-economical N = 10	*p*	Economical N = 9	Non-economical N = 10	*p*
Energy cost of running									
Energy cost of running (J kg^−1^ m^−1^)	2.15 ± 0.20	2.93 ± 0.42	** < .001**	3.52 ± 0.18	4.20 ± 0.32	** < .001**	5.82 ± 0.27	6.55 ± 0.42	** < .001**
Running kinematics and GRF									
Contact time (s)	0.28 ± 0.03	0.29 ± 0.03	0.697	0.30 ± 0.04	0.28 ± 0.02	0.199	0.30 ± 0.02	0.28 ± 0.02	**0.013**
Aerial time (s)	0.10 ± 0.03	0.09 ± 0.02	0.621	0.07 ± 0.02	0.09 ± 0.02	0.063	0.06 ± 0.01	0.07 ± 0.02	0.130
Duty factor (%)	74.1 ± 8.9	75.7 ± 6.5	0.654	80.5 ± 5.8	76.0 ± 5.3	0.093	83.7 ± 4.1	79.5 ± 5.5	0.080
Step frequency (Hz)	2.65 ± 0.09	2.66 ± 0.12	0.900	2.71 ± 0.16	2.70 ± 0.09	0.864	2.75 ± 0.10	2.86 ± 0.10	**0.026**
Step length (m)	1.05 ± 0.04	1.05 ± 0.05	0.932	1.03 ± 0.06	1.03 ± 0.03	0.947	1.01 ± 0.04	0.97 ± 0.03	**0.038**
Relative step length (% of height)	60.1 ± 2.4	59.8 ± 4.8	0.871	58.3 ± 3.3	59.3 ± 4.4	0.560	56.5 ± 3.5	56.8 ± 3.2	0.854
Peak vertical GRF (kN)	1.43 ± 0.14	1.39 ± 0.2	0.619	1.36 ± 0.16	1.34 ± 0.22	0.875	1.34 ± 0.14	1.25 ± 0.2	0.252
Mass-specific vertical GRF (N kg^−1^)	21.1 ± 2.5	20.5 ± 1.7	0.573	19.2 ± 1.5	20.4 ± 1.4	0.102	18.5 ± 0.9	19.5 ± 1.3	0.075
Mechanical work									
External work (J kg^−1^)	3.13 ± 0.47	3.00 ± 0.43	0.537	2.69 ± 0.23	2.81 ± 0.34	0.368	2.71 ± 0.14	2.80 ± 0.23	0.317
Positive external work (J kg^−1^)	1.11 ± 0.25	1.04 ± 0.22	0.502	1.38 ± 0.12	1.45 ± 0.18	0.363	1.87 ± 0.08	1.93 ± 0.13	0.255
Negative external work (J kg^−1^)	− 2.02 ± 0.23	− 1.97 ± 0.21		− 1.30 ± 0.11	− 1.36 ± 0.17	0.375	− 0.84 ± 0.06	− 0.87 ± 0.11	0.468
Percentage negative external work (%)	64.9 ± 2.5	65.7 ± 2.1	0.448	48.5 ± 0.2	48.5 ± 0.2	0.652	30.8 ± 0.8	30.8 ± 1.3	0.996

*GRF* peak ground reaction force. Significant values are in [bold].

## Discussion

This study provides new insights into biomechanical factors according to the level of metabolic economy of runners on different running slopes. The main findings of the present study are that (1) aerial time, peak vertical ground reaction force, positive mechanical work, and duty factor were associated with Cr during LR and UR; while contact time correlated only with Cr during UR; (2) no relationship was observed between Cr and biomechanical responses during DR; and (3) most economical runners tend to have specific running pattern adaptations (i.e., longer contact time and greater step length as well as a lower step frequency) during UR only. These results partially support the hypothesis since specific running pattern responses were characterized by a lower metabolic cost of running on flat or positive slopes but not on negative slope.

During UR, Cr positively correlated with several running pattern parameters such as W_ext_^+^, aerial time, and mass specific GRF, and negatively correlated with contact time and duty factor. These correlations demonstrate that UR is related to specific running pattern parameters and highlight that the running pattern may influence Cr, especially during UR. Our results revealed that Cr, W_ext_^−^, W_ext_^+^, duty factor, and step frequency were lower; whereas areal time, step length (in absolute and relative to the height), GRF (expressed in both absolute and relative values), and the percentage of the W_ext_^−^ were higher during LR than during UR, which is rather consistent with the literature^23^. Furthermore, as already observed on similar slopes^8,9,34^, the running economy was reduced when running on the positive slope.

Most of the mechanical work performed comes from positive external mechanical work during UR (~ 69% of W_ext_ was provided by W_ext_^+^ and only ~ 31% by W_ext_^−^; Table 2). Comparable distribution on equivalent slope and speed was reported^9^. These results confirm that UR is primarily a concentric muscle contraction that is energy-consuming^25^. Indeed, the W_ext_^+^ represents the amount of mechanical energy spent during the pushing phase to elevate and move forward the CoM^24^.

It has been highlighted that the increase in W_ext_^+^ was caused by the elevation of CoM related to the upward movement of the body during UR^24^. The external work is the product of vertical displacement of the CoM and the step frequency. It was observed that lower vertical displacements of the CoM and/or a higher step frequency in LR were associated with better running economy^35^. Therefore, one could potentially expect a similar relationship during UR. However, no correlation was observed between step frequency and Cr, possibly because runners choose their own optimal step frequency and step length, whatever the slope level^16^, close to their minimal Cr^36^. Nevertheless, other spatiotemporal parameters such as the contact time and aerial time were correlated with Cr during UR. Contact time was negatively correlated to the Cr, meaning that a longer contact was associated with a better running economy. This result is in agreement with the observation made by Vernillo et al.^37^ after an ultramarathon event (330 km with 24,000 m elevation gain). The UR Cr (4 min at 6 km h^−1^ and + 15% incline) was negatively correlated with contact time, duty factor, and as well as rate of force application (characterized by inverse contact time: t_c_^−1^). For instance, shorter contact time reduces the time allowed to generate force into the ground and increases the rate at which the muscle fibers shorten^38,39^, so more fast muscle fibers or muscle mass should be required for a given applied force^37,38^. The step frequency depends on the interrelationship between contact time and step length. Thus, for a given step frequency, increasing the aerial time will shorten the contact time and lead to Cr deterioration, since the metabolic cost of force generation increases as the contact time shortens^38^. In addition, as it exists an inverse relationship between the step frequency and the vertical oscillation of CoM in running^35^, Furthermore, reducing aerial time and vertical displacement (which will occur together) are the result of less external positive work (resulting in a reduced vertical velocity at takeoff) during UR. However, an excessive increase of the stride frequency during running to reduce mechanical work could be disadvantageous as it causes a Cr raise^16^. The negative correlation observed between Cr and the duty factor might underline the importance of optimizing energy transfer (from metabolic to mechanics) to reduce energy demands in UR. Altogether, increasing the stance phase and decreasing the aerial time may be an appropriate strategy for improving running economy during UR. As such, patterns of locomotion may play a decisive role in lowering the cost of locomotion by walking compared to running on a steep positive slope^40^. Indeed, walking pattern is characterized by a longer contact time, a higher duty factor, and a lower stride frequency associated with reduced muscle activation compared to the running pattern on a 30° slope^41^.

The present data showed a relationship between the Cr and GRF. Normalized GRF to body weight was positively correlated with Cr during UR. Since GRF is the result of the forces produced by all the muscles in the vertical direction during the stance phase, an excessive GRF value in this orientation is a waste of energy. Thus, minimizing the vertical GRF seems to be more economical during UR. Increasing step frequency could lower the vertical GRF and might be a useful strategy in UR. Therefore, the adoption of strategies to reduce vertical GRF forces should be incorporated in training programs in order to improve running economy during UR as well as during LR. However, such adjustments must be individually adapted.

In the present study, we confirm that negative slope has a significant effect on the running pattern and decreases Cr compared to LR^42^. Total mechanical work, W_ext_^−^, proportion of W_ext_^−^, aerial time, and GRF (expressed in absolute and mass-specific values), increased in DR while the W_ext_^+^ decreased, in agreement with the literature^9,23,24,43^. However, conversely to UR, downhill Cr was not correlated with any mechanical aspects, suggesting that, at least for the present velocity and slope, there was not a more economical running pattern during DR. These results are not in agreement with previous results^12^ which reported a significant correlation between Cr and several spatiotemporal parameters such as step length, step frequency, and contact time during DR (− 15%). According to these later results, it was suggested that the ability to store and restitute elastic energy had an important role in DR Cr^12^. The difference observed in the literature may come from differences in the experimental design and the fitness level of the participants. As observed by Minetti et al.^9^ more than half (~ 65%) of the total external work is provided by W_ext_^−^. The latter represents the work done during the braking phase of the stance phase. During this phase, the knee extensor muscles forcibly lengthen (i.e., eccentric muscle action) under the potential effect of gravity to limit the drop-down of the CoM. From an energetical point of view, this eccentric muscle’s action requires less energy than a concentric muscle contraction^25^, and part of the potential energy from the vertical oscillation of the CoM is either dissipated as wasted heat (mostly) or stored in the muscle–tendon units during the braking phase prior its restitution during the pushing phase. The stretch–shortening cycle is mainly involved during DR^16,44^ and is known to be less energy consuming than purely concentric actions^45^, saving energy and reducing the Cr as well^45^. For moderate negative slopes (~ 15%), the elastic energy stored in the muscle–tendon units can supply almost all the energy demand for the push phase^44^. However, no correlation was observed between W_ext_^−^ and the Cr.

Runners have their own running style based on ability and experience which implies that a similar Cr from one individual to another can be associated with different biomechanical parameters. Indeed, there was only a relatively small influence of each of the parameters measured on CR, even when the relationship was significant (Fig. 1). Therefore, we have to be cautious when suggesting potential gait modifications for performance enhancement. Changing one parameter of the running pattern can alter the overall mechanics and potentially the running economy^46^. For example, ± 15% changes in preferred step frequency increased Cr by ~ 20% in DR^16^. Running in negative slopes is demanding for the body, as greater braking force must be applied on the ground to maintain a constant speed, which can generate muscle damage^47^. Furthermore, since force absorption is less energetically demanding, runners may neglect their running mechanics. A more protective running pattern is privileged by runners based on their experience in DR^9^. Running on negative slopes where the fear of falling is higher could also exacerbate emotional aspect, when compared to LR and UR. During treadmill running, irrespective of the slope, it is well-known that the mechanics is different than during overground running: the influence of the motion belt that affects both potential and kinetic works remains difficult to be accurately assessed^48^. Therefore, we have to be cautious for translating the present findings to field running.

The present study compared the metabolic and biomechanical responses of economical and non-economical runners at different slopes. Individual Cr values in the three slope conditions were used to split participants into two groups. We showed that the most economical runners remained the same ones, independently of the slope (i.e., in DR, LR or UR). This result is rather consistent with the literature: Willis et al.^7^ reported a strong correlation between Cr measured in LR and in UR (12% slope) in a group of elite ultra-trail runners (6 males and 5 females), while Balducci et al.^49^ found no correlation between LR and UR (12.5 or 25% slope) in trained trail runners^49^. Moreover, in the present study, there were differences in biomechanical responses between the two groups, but only in UR economical runners had longer contact time and step length compared to less economical runners, while their step frequency was smaller. Ultimately, the longer duration of the stance phase may allow runners to optimize the direction of propulsive force and the time allowed to apply force to the ground^50^. Indeed, mass-specific GRF tends to be lower in the economical runners than in the non-economical group (*p* = 0.070; Table 3), suggesting that lower mass-specific GRF in UR may allow to reduce the metabolic cost of running. A lower vertical force during the stance phase was observed for the group of runners who had the lowest oxygen consumption for a given speed (~ 13 km h^−1^) on flat terrain^17^.

No difference for all the biomechanical variables was observed in DR and LR between the groups of economical and non-economical runners, i.e., with different Cr. This result is rather in line with the literature^13,14^, and may be partly explained by the heterogeneity of the population in the present study. Less experienced runners tend to have a greater stride to stride variability than experienced runners^51^. The Cr may be influenced by many other factors, such as anthropometry, flexibility, and joint kinematics but also by physiological differences (e.g., metabolic efficiency) or equipment (e.g., running shoes)^18^. Runners naturally chose their optimal running pattern themselves to minimize their Cr^13^. Each one having its own specificity, the number of mechanical combinations is likely very important. Nevertheless, even if two groups use different running strategies, there were no significant differences in Cr^13,14^.

## Conclusions

The present study reported that Cr was related to few key running pattern parameters (i.e., contact time, aerial time, mass specific GRF and positive mechanical external work) mainly in UR, but not in DR. Moreover, all running pattern parameters were similar between economical and non-economical runners in DR and LR, but not in UR. Interestingly, the contact time and the step length were longer, whereas the step frequency was lower in the group of economical runners compared to the group of non-economical runners in UR. These results provide interesting insights concerning an optimal running pattern to reduce the cost of locomotion, and consequently improve performance during graded running. In practice, it may be preferable to reduce step frequency, or even to shift to walking, on a positive slope to increase step length and slow down the knee extension during the propulsive phase. On steep slopes, poles could facilitate this mechanism^52^. Overall, the present study emphasizes that the mechanics of LR and UR are fundamentally different. Future investigations are needed to deepen the knowledge with a heterogeneous population (trained or untrained runners) to improve the training protocols of mountain or trail runners.

## Data Availability

The datasets used and/or analyzed during the current study are available from the corresponding author on reasonable request.
